# Clinical analysis of 45 cases of perforation were identified during endoscopic retrograde cholangiopancreatography procedure

**DOI:** 10.3389/fmed.2022.1039954

**Published:** 2022-11-24

**Authors:** Yin-Shui Miao, Yuan-Yuan Li, Bo-Wen Cheng, Yan-Fang Zhan, Sheng Zeng, Xiao-Jiang Zhou, You-Xiang Chen, Nong-Hua Lv, Guo-Hua Li

**Affiliations:** ^1^Department of Gastroenterology, The First Affiliated Hospital of Nanchang University, Nanchang, Jiangxi, China; ^2^School of Medicine, Nanchang University, Nanchang, Jiangxi, China; ^3^Department of Record Room, The First Affiliated Hospital of Nanchang University, Nanchang, China

**Keywords:** endoscopic retrograde cholangiopancreatography (ERCP), evaluation, perforation, strategy, duodenal perforations

## Abstract

**Background:**

Endoscopic retrograde cholangiopancreatography (ERCP) has become an important method to diagnose and treat biliary-pancreatic diseases. Perforations are infrequent but serious complications can occur during ERCPs. However, it is unclear which patients are suitable for surgery and when these patients should receive surgery.

**Aim:**

To analyze the outcome of 45 patients with endoscopic retrograde cholangiopancreatography (ERCP) related perforation.

**Materials and methods:**

We retrospectively reviewed all 45 patients with ERCP-related perforation between January 2003 and December 2017, and observed the location and causes of perforation, treatment strategies, and mortality.

**Results:**

Twenty thousand four hundred and seventy-nine patients received ERCP procedures from January 2003 to December 2017 in our digestive endoscopy center. Forty-five patients suffered from ERCP-related perforations. The incidence rate of ERCP-related perforations was 0.22%. Twenty-six patients suffered from periampullary perforations, 15 patients suffered from duodenal wall perforations, 1 patient suffered from a fundus perforation, 1 patient suffered from a residual gallbladder duct perforation, 1 patient suffered from a papillary diverticulum perforation, and 1 patient suffered from an intrahepatic bile duct perforation. Six patients with duodenal perforations underwent surgery, and the other patients received conservative treatment. One patient with a duodenal perforation and ERCP-related pancreatitis died of heart failure, and all the other patients recovered. The mortality rate was 2.2%.

**Conclusion:**

Endoscopic closure is seen as the first method for treating Stapfer type I perforations in the early phase, and surgery is seen as a remedial method when local treatment was failed. The Stapfer type II to type IV perforations can recover by conservative treatment.

## Introduction

Endoscopic retrograde cholangiopancreatography (ERCP) has become an important method to diagnose and treat biliary-pancreatic diseases. Perforations are infrequent but serious complications can occur during ERCPs. Multiple case series have shown the overall risk of perforation during ERCP to be < 1%, with a mortality range of 7.8–9.9% (1–5). Many patients with ERCP-related perforations recovered by undergoing surgery or conservative therapy (6–10). However, it is unclear which patients are suitable for surgery and when these patients should receive surgery. In this study, we evaluated our experiences in the management of ERCP-related perforations at our digestive endoscopy center. We now report the results we found.

## Materials and methods

### Patients

We collected cases at our endoscopy center (The Digestive Endoscopy Center of Jiangxi Province) from January 2003 to December 2017. We retrospectively reviewed all cases in this period. The patients’ demographics, including age, sex, and comorbidities, such as coronary heart disease (CHD), chronic obstructive pulmonary disease (COPD), chronic renal failure, and malignancies, were investigated. The indication for ERCP, clinical presentation of perforation, and management were also recorded and analyzed.

### Anesthesia and surgery

Before the ERCP, a routine preoperative blood examination, electrocardiography, chest X-ray, and echocardiography were conducted. On the day of the ERCP, the patient took medicine that treats hypertension and coronary heart disease in the morning before the operation. The fasting blood glucose of diabetic patients was controlled at 8–10 mmol/L. The patients fasted for 8 h before surgery and signed consent for the ERCP. Intravenous anesthesia was administered with propofol, a TJF-240, or JF-240(Olympus, Tokyo, Japan). Duodenoscope was used for endoscopy, and third-generation visualization was achieved with a contrast agent (iodide injection, Guerbet).

Angiography was performed after selective intubation to understand the nature of the biliary and pancreatic duct lesions with different processing methods. For cases with a combination of clinical circumstances, such as a diagnosis of common bile duct stones in the lining of the duodenal papilla sphincter incision (EST), operations such as balloon lithotomy were performed on the stones; in cases of inflammatory bile duct stenosis or ampullary tumors, endoscopic biliary stent implantation (ERBD), or nasobiliary drainage (ENBD) procedure was performed. For Oddi sphincter dysfunction, a duodenal papillary sphincterotomy was performed.

### Postoperative treatment

Postoperative treatment included fasting for 24 h after surgery, acid inhibition, rehydration, other symptomatic treatments, and if needed, antibiotic treatment. Abdominal pain, haematemesis, melena, fever, and other conditions were observed, and blood amylase tests and routine blood tests were performed at 3 and 24 h after surgery.

## Results

A total of 20,479 ERCPs were performed at our endoscopy center. A total of 45 cases with ERCP-related perforations (0.22%) were identified. The 45 cases with ERCP-related perforations (45/20,479) were identified by X-ray and/or duodenoscopy during the ERCP. The average age of the patients was 67 ± 12.6 years old (from 25 to 88 years old); the patients included 18 male patients and 27 female patients. The incidence of ERCP-related perforations was 0.22%. The demographical characteristics and clinical data of these patients are presented in Table 1.

**TABLE 1:** Demographical characteristics and clinical data of the 45 patients.

Variables	
**Demographics**	
Total Patients	20479
Age (y, Mean ± SD)	63 ± 14.8 (from 10 to 90 years old)
Male-to-Female ratio	11873:8607
ERCP-related perforation	45
Age (y, Mean ± SD)	67 ± 12.6 (from 25 to 88 years old)
Male-to-Female ratio	18:27
**Indication for ERCP**	
Choledocholithiasis	44
Hilar cholangiocarcinoma	1
**Comorbidities**	
Acute pancreatitis	5(11.11%)
Hypertension	5(11.11%)
Billroth II gastrectomy	4(8.89%)
Chronic obstructive pulmonary diseases	3(6.67%)
Diabetes mellitus	2(4.45%)
Coronary heart disease	1(2.22%)
Coronary heart disease and chronic obstructive pulmonary disease	1(2.22%)
Arthrolithiasis and chronic obstructive pulmonary disease	1(2.22%)

Among the 45 perforations, 26 patients experienced peri-ampullary perforations, 15 patients experienced duodenal wall perforations, including 3 afferent limb perforations, and the other patients experienced peri-ampullary diverticulum perforations, small bile duct perforations on the liver surface, residual duct of gallbladder perforations, and fundus perforations. The fundus perforation and 15 duodenal wall perforations resulted from duodenoscopy; the peri-ampullary diverticulum perforation, the residual duct of gallbladder perforation, and five peri-ampullary perforations resulted from the stone extraction basket; 15 peri-ampullary perforations resulted from papillotomy; 4 peri-ampullary perforations resulted from precut surgical methods; 2 peri-ampullary perforations resulted from balloon catheter dilation; and the small bile duct perforation on the liver surface resulted from the guide wire. The Stapfer types, scope, and etiology of perforations are listed in Table 2.

**TABLE 2:** The Stapfer type, scope and mechanism of ERCP-related perforations.

Stapfer type	Perforation scope	Amount	Mechanism
Stapfer I	duodenal wall	15	Duodenoscopy
Stapfer I	peri-ampullary diverticulum	1	Basket
Stapfer I	gasric fundus	1	Duodenoscopy
Stapfer II	peri-ampullary	26	Papillotomy, basket, pre-cut and balloon catheter dilation
Stapfer III	small bile duct on the liver surface	1	guide wire
Stapfer III	residual duct of gallbladder	1	Basket

**FIGURE 1:**
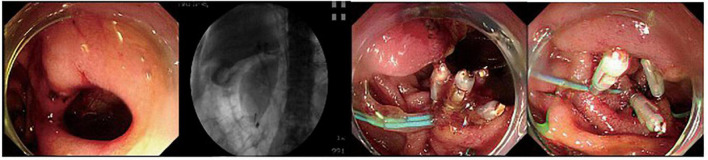
A duodenal wall perforation closed by clips and nylon rope under a single cavity forward-viewing endoscope. The perforation had only signs of retroperitoneal perforation.

**FIGURE 2:**
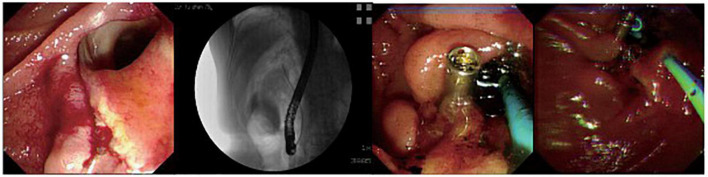
A patient with peri-ampullary perforation received ERBD and ERPD. The perforation had signs of retroperitoneal and peritoneal perforation.

All ERCP-related perforations had been diagnosed during the ERCP procedure. The presentation of a retroperitoneal perforation showed skin emphysema and a clear kidney shadow in fluoroscopy X-ray even individual cases developing pneumoscrotum (the gas could reach the scrotum by tracking along the transversalis fascia, which forms the innermost covering layer of the spermatic cord) (11), and the presentation of a peritoneal perforation showed a free gas shadow under the diaphragm in fluoroscopy X-ray, a visible gastrointestinal wall lesion under the endoscope, and signs of peritonitis. Most duodenal wall perforations had signs of peritoneal perforations and most peri-ampullary perforations had signs of retroperitoneal perforations. However, two peri-ampullary perforations had both intraperitoneal and retroperitoneal perforation manifestations, and a duodenal perforation and peri-ampullary diverticulum perforation had only retroperitoneal perforation manifestations (Figures 1, 2).

**FIGURE 3:**
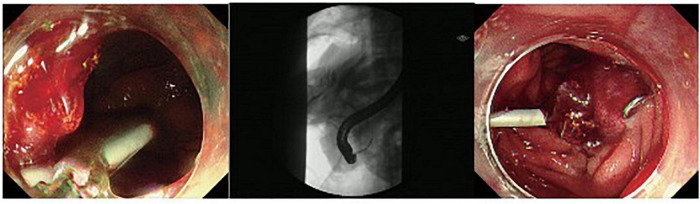
A duodenal wall perforation closed by OTSC. The patient received ERBD. The perforation per had signs of peritoneal perforation.

**FIGURE 4:**
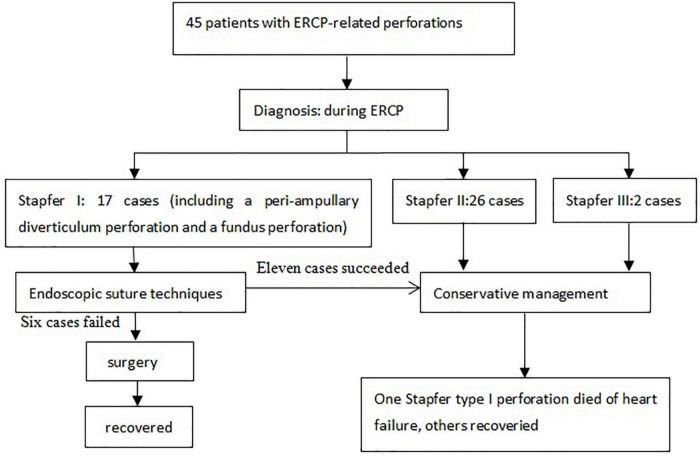
The management and outcome of 45 patients with ERCP-related perforation.

The fundus perforation, the peri-ampullary diverticulum perforation, and eight duodenal perforations were treated by closing the lesion, performing endoscopic nasobiliary drainage (ENBD) or endoscopic retrograde biliary drainage (ERBD), conducting gastrointestinal decompression, and using proton pump inhibitor (PPI), somatostatin (SS), and broad-spectrum antibiotics for 5–7 days. Each lesion was closed by clips, purse string sutures, or over-the-scope-clip (OTSC) (Figures 1–3). Three afferent limb perforations and three duodenal wall perforations were treated through surgery. The small bile duct perforation on the liver surface, the residual duct of gallbladder perforation, and the 26 peri-ampullary perforations were healed through nasobiliary drainage or biliary stenting drainage, gastrointestinal decompression, and using PPI, SS, and broad-spectrum antibiotics for 5–7 days. Biliary stents are typically 8.5 Fr × 7 cm in size, whereas pancreatic stents are typically 5 Fr × 5 cm in size. If no unusual conditions exist, the stents will be removed after 1 month of satisfactory drainage. Three patients received endoscopic retrograde pancreatic drainage (ERPD) at the same time. The 81-year-old female patient with a duodenal wall perforation, which had been closed with OTSC, died of heart failure and post-ERCP pancreatitis 3 days after the ERCP procedure. The other patients recovered successfully (Figure 4). Management outcomes of the 45 patients were summarized in Table 3. The mortality was 2.2% (1/45).

**TABLE 3:** Management outcomes of the 45 patients.

		Treatment methods (N)			Outcome (N)		
		Conservative treatment			Surgery	Recoveried	Died
		ENBD/ERBD	ENBD/ERBD + ERPD	Closure + ENBD/ERBD			
Perforation types	Stapfer I	0	0	11	6	16	1
	Stapfer II	23	3	0	0	26	0
	Stapfer III	2	0	0	0	2	0
Total		25	3	11	6	44	1

ENBD, endoscopic nasobiliary drainage; ERBD, endoscopic retrograde biliary drainage; ERPD, endoscopic retrograde pancreatic drainage.

## Discussion

Perforation related to ERCP is the most serious complication to avoid because it can potentially threaten the life of patients. Consequently, studies regarding this complication should be necessary; however, there have been only a few reports with a limited number of cases, mainly owing to its rarity. Table 4 is a compilation of relevant material produced since the 20th century (a MEDLINE search was performed from 2000–2022 using the keywords “perforation”, “ERCP”, and “endoscopic sphincterotomy”). Reviewing 17 studies, including 140,588 patients, the incidence was 0.33% (95% CI: 0.30–0.36). The overall mortality was 8.8% (95% CI: 6.21–11.35). According to Stapfer classification, type I counted 27.6%, type II counted 47.5%, and type III counted 19.1%. In our study, 16 perforations resulted from duodenoscopy injuries, 15 peri-ampullary perforations resulted from papillotomy, 4 peri-ampullary perforations resulted from precut surgical methods, 7 perforations resulted from inserting the basket into the common bile duct (CBD) after papillotomy while removing the stone, 2 peri-ampullary perforations resulted from papilla balloon dilation, and 1 resulted from the guide wire passing through the liver surface. The mechanism of injury is mentioned in 573 patients from 20 studies (Table 5). Endoscopic sphincterotomy was responsible for 41.1% of perforations, insertion, and manipulations of the endoscope for 25.2%, guide wires for 14.6%, dilation of strictures for 2.9%, other instruments for 6.8%, stent insertion or migration for 1.9%, and in 7.6% of cases, the etiology was unknown. This showed that endoscopic sphincterotomy (EST) was the most prevalent cause of ERCP-related perforations, followed by endoscopic insertion and guide wire.

**TABLE 4:** In 17 papers, the incidence of ERCP-related perforation, overall mortality, and treatment were reviewed.

Author	*n*	Perforations	Stapfer Types (surgery/died)				Surgery treatment	Mortality	
			I	II	III	IV		After surgery	Overall
Stapfer et al. (3)	1413	14 (0.35%)	5 (5/2)	6 (3/0)	3 (1/0)		9	2	2 (14%)
Enns et al. (21)	9314	33 (0.35%)	5 (4/0)	13 (2/0)	15 (1/0)		7	0	1 (3%)
Wu et al. (22)	6620	30 (0.45%)	5 (4/1)	11 (5/4)	7		11	5	5 (16%)
Assalia et al. (23)	3104	22 (0.70%)	2 (2/0)	17 (2/1)	2		4	1	1 (4.5%)
Fatima et al. (24)	12427	75 (0.60%)	8(7/3)	26 (12/0)	35(3/0)	6	22	3	5 (6.6%)
Mao et al. (25)	2432	9 (0.37%)	8 (3/0)	1			3	0	0
Avgerinos et al. (26)	4358	15 (0.34%)	9 (9/0)	3 (3/1)		1	14	3	3 (20%)
Morgan et al. (27)	12817	24 (0.18%)	12 (10/1)	12			12	1	1 (4.1%)
Kim et al. (28)	7638	13 (0.17%)	4 (3/0)	5 (3/0)	4 (1/0)		7	0	0
Polydorou et al. (29)	9880	44 (0.44%)	7 (6/2)	30 (6/0)	5	2	12	2	2 (4.5%)
Dubecz et al. (30)	12232	11 (0.08%)	7 (4/0)	3	1		4	0	2 (18%)
Kwon et al. (31)	8381	53 (0.63%)	21 (17/1)	32 (1/0)			18	1	3 (5.6%)
Li et al. (32)	8504	16 (0.45%)	7 (4/0)	5	4		4	0	0
Jin et al. (7)	22998	59 (0.26%)	17 (11/3)	36 (7/0)	6		18	3	5 (8.4%)
Miller et al. (8)	1638	27 (1.6%)	5 (5/2)	12 (11/6)	5	5	16	8	9 (33%)
Kodali et al. (33)	8264	12 (0.15%)	2 (2/0)	8 (3/0)	2		5	0	0
Cakar et al. (34)	8568	10 (0.12%)	5 (4/2)	2		3 (2/0)	6	2	2 (20%)
Total	140588	467 (0.33%)	129 (100/17)	222 (58/12)	89(6/0)	17 (2/0)	172	31	41 (8.8%)

**TABLE 5:** The etiology of ERCP-related perforation is described in 20 studies.

Author	Endoscope	EST	Guide wire	Dilation of strictures	Other instrument	Stent insertion or migration	Unknown	Total
Stapfer et al. (3)	5	6	3					14
Enns et al. (21)	5	13	13	2				33
Kayhan et al. (35)	2	15						17
Wu et al. (22)	5	11	7				7	30
Assalia et al. (23)	2	17	2			1		22
Fatima et al. (24)	8	11	24	5	9	7	11	75
Knudson et al. (36)	6	11			4	3	8	32
Mao et al. (25)		8			1			9
Avgerinos et al. (26)	9	3					3	15
Morgan et al. (27)	12	12						24
Krishna et al. (37)	11	1	2					14
Kim et al. (28)	4	3	4		2			13
Polydorou et al. (29)	7	30	2	2	3			44
Kim et al. (38)	13	25	23		2		5	68
Dubecz et al. (30)	7	3	1					11
Kwon et al. (31)	21	24	2	6				53
Li et al. (32)	7	5			4			16
Alfieri et al. (13)	6	15	1				8	30
Jin et al. (7)	17	22		2	15		3	59
Kodali et al. (33)	2	8	2					12
Total	149 (25.2%)	243 (41.1%)	86 (14.6%)	17 (2.9%)	40 (6.8%)	11 (1.9%)	45 (7.6%)	591

EST, endoscopic sphincterotomy.

According to the AGA 2021 updated expert review, delayed recognition of a perforation more than 6 h after ERCP is associated with an increased length of hospital stay and mortality and may result in a more complicated surgical intervention (5, 7, 9). Thus, the early diagnosis of the complication is very important. We should especially note the signs of retroperitoneal and peritoneal perforations (12). In our study, all perforations were diagnosed during the ERCP procedure by X-ray fluoroscopy and/or endoscopy. We typically performed fluoroscopy for each patient before and after the ERCP procedure to determine whether a perforation occurred during the procedure. This habit helped us to diagnose these perforations early, which may be the reason for a lower mortality rate in our study.

After the recognition of an ERCP-related perforation, the first dilemma is conservative treatment or surgery (6, 13, 14). A total of 172 of the 467 perforations underwent surgery, with 31 deaths, for a surgical rate of 36.8%, and a postoperative mortality rate of 18%, with surgical deaths accounting for 75.6% of total deaths. Non-operative therapy was given to 295 patients, with 10 fatalities and a mortality rate of 3.4%, accounting for 24.3% of all deaths.

For Stapfer type I perforations, there were 117 cases in the 17 papers we reviewed. Of these cases, 100 underwent surgery and 17 died. The postoperative mortality rate of 17% remains still high. In our study, one fundus perforation and nine duodenal perforations (including one peri-ampullary diverticulum perforation) were closed successfully by clips, purse string sutures, or OTSC and treated by conservative treatment. Seven duodenal perforations (including three afferent limb perforations) were treated by surgery. An 81-year-old female patient with COPD and coronary heart disease had a duodenal perforation, which was closed by OTSC, and ERCP-related pancreatitis and died of heart failure 3 days after the ERCP procedure. The other patients recovered successfully. This outcome is consistent with the AGA expert review’s conclusion that for patients who do undergo successful endoscopic closure, the chance of clinical successful recovery without surgery is > 90% (5, 15). Therefore, we recommend endoscopic closure is seen as the first method for treating Stapfer type I perforations in the early phase, and surgery is seen as a remedial method when local treatment fails (16, 17).

For Stapfer type II perforations, most were successfully treated conservatively, with only 58 cases undergoing surgery and 12 postoperative deaths. The operative rate was 26.1% and the postoperative mortality rate was 20.7%. In our study, all peri-ampullary perforations in the early phase recovered successfully with conservative treatment, including nasobiliary or biliary stenting drainage, gastrointestinal decompression, fasting, intravenous nutrition, and using PPI, SS, and broad-spectrum antibiotics for 5–7 days. Our experiences suggest that these peri-ampullary perforations could recover with these conservative treatment methods in the early phase, which may be because (i) the peri-ampullary perforations were small perforations; and (ii) conservative management in the early phase could alleviate the stimulation and secretion of gastric acid, bile, and pancreatic liquid. This result was in line with the recent statement by ESGE and AGA that a majority of patients with Stapfer type II perforations can be managed non-surgically, with emergency surgery indicated only in rare cases where a major contrast leakage is insufficiently sealed (2, 5).

The perforations that are classified as Stapfer types III and IV should be treated by conservative treatment because all patients with type III or type IV perforations recovered by conservative treatment in recent reports (8–10, 12, 15, 18–20). Moreover, effective therapy should also include preventing or treating infections using broad-spectrum antibiotics.

In our study, we have minimal experience treating ERCP-related perforations in the late phase, which can present large fluid exudation and infection in the retroperitoneal space and peritoneal cavity. According to the statement issued by ESGE in 2020, regional management of drained collections is required. This can be performed through percutaneous access or during surgery, which also allows the evacuation of debris (2). However, the statement does not specify which patients require surgery and when these patients should receive surgery. In general, a surgical operation might increase the risk of trauma or death and should be applied cautiously. When a large lesion could not be closed or the fluid exudation and infection in the retroperitoneal space and peritoneal cavity could not be adequately drained, surgery may be necessary (16, 17). In addition, percutaneous drainage merits additional investigation.

## Conclusion

Endoscopic sphincterotomy (EST) was the most prevalent cause of ERCP-related perforations, followed by endoscopic insertion and guide wire. Endoscopic closure is seen as the first method for treating Stapfer type I perforations in the early phase, and surgery is seen as a remedial method when local treatment fails. Patients with Stapfer type II to type IV perforations could recover by undergoing conservative treatment.

## Data availability statement

The raw data supporting the conclusions of this article will be made available by the authors, without undue reservation.

## Ethics statement

The studies involving human participants were reviewed and approved by Ethics Committee of The First Affiliated Hospital of Nanchang University. The patients/participants provided their written informed consent to participate in this study.

## Author contributions

Y-SM and Y-YL designed the study, performed the data analysis, wrote the manuscript, and interpreted the results. X-JZ, Y-XC, and SZ extracted the data and revised the manuscript. N-HL and Y-FZ designed the study and interpreted the results. G-HL and B-WC were involved in intellectual content, designed the study, and interpreted the results. All authors read and approved the final manuscript.
